# Restricted Use of Erythropoiesis-Stimulating Agent is Safe and Associated with Deferred Dialysis Initiation in Stage 5 Chronic Kidney Disease

**DOI:** 10.1038/srep44013

**Published:** 2017-03-08

**Authors:** Szu-Yu Pan, Wen-Chih Chiang, Ping-Min Chen, Heng-Hsiu Liu, Yu-Hsiang Chou, Tai-Shuan Lai, Chun-Fu Lai, Yen-Ling Chiu, Wan-Yu Lin, Yung-Ming Chen, Tzong-Shinn Chu, Shuei-Liong Lin

**Affiliations:** 1 Division of Nephrology, Department of Internal Medicine, National Taiwan University Hospital, Taipei, Taiwan; 2 Department of Internal Medicine, Far Eastern Memorial Hospital, New Taipei City, Taiwan; 3 Graduate Institute of Physiology, National Taiwan University College of Medicine, Taipei, Taiwan; 4 Institute of Epidemiology and Preventive Medicine, National Taiwan University College of Public Health, Taipei, Taiwan; 5 Department of Internal Medicine, National Taiwan University Hospital Bei-Hu Branch, Taipei, Taiwan; 6 Department of Public Health, National Taiwan University College of Public Health, Taipei, Taiwan; 7 Department of Integrated Diagnostics & Therapeutics, National Taiwan University Hospital, Taipei, Taiwan; 8 Research Center for Development Biology and Regenerative Medicine, National Taiwan University, Taipei, Taiwan

## Abstract

The effect of erythropoiesis-stimulating agent (ESA) on dialysis initiation in advanced chronic kidney disease (CKD) patients is not clear. We retrospectively analyzed the outcome of dialysis initiation in a stage 5 CKD cohort with ESA reimbursement limited to the maximal standardized monthly ESA dose equivalent to epoetin beta 20,000 U by the National Health Insurance program. Totally 423 patients were followed up for a median of 1.37 year. A time-dependent Cox regression model, adjusted for monthly levels of estimated glomerular filtration rate (eGFR) and hemoglobin, was constructed to investigate the association between ESA and outcome. The standardized monthly ESA dose in ESA users was 16,000 ± 3,900 U of epoetin beta. Annual changes of hemoglobin were −0.29 ± 2.19 and −0.99 ± 2.46 g/dL in ESA users and ESA non-users, respectively (P = 0.038). However, annual eGFR decline rates were not different between ESA users and non-users. After adjustment, ESA use was associated with deferred dialysis initiation (hazard ratio 0.63, 95% confidence interval 0.42–0.93, P = 0.021). The protective effect remained when the monthly ESA doses were incorporated. Our data showed that restricted use of ESA was safe and associated with deferred dialysis initiation in stage 5 CKD patients.

Chronic kidney disease (CKD) is highly prevalent worldwide and contributes to a heavy health care burden^1^,^2^,^3^,^4^,^5^. Finding means to halt the progression of CKD and reduce the burden of end-stage renal disease (ESRD) is of paramount clinical importance.

Experimental studies have highlighted the promise of renoprotective effect of exogenous erythropoietin (EPO) therapy in various CKD models^6^, including remnant kidney^7^, diabetic nephropathy^8^, ischemia-reperfusion injury^9^, and chronic allograft injury of transplant kidney^10^. Interestingly, most studies adopted a dose of erythropoiesis-stimulating agent (ESA) as low as not to elevate the hemoglobin (Hb) level. Mechanistically, activation of β-common receptor (βcR) and downstream Akt or JAK2-STAT5 pathways were implicated^6^,^11^,^12^. Importantly, the βcR was shown to be dispensable for erythropoiesis^13^.

The results of clinical trials on the renoprotective effect of ESA are less encouraging. Small randomized clinical trials (RCTs) showed benefits in retarding progression of CKD and allograft nephropathy^14^,^15^, while large scale RCTs failed to demonstrate benefit^16^,^17^. Notably, although ESA treatment significantly increased the Hb levels in all studies, much higher doses were adopted in studies failing to show benefits of ESA^16^,^17^. Besides, secondary analyses of these trials implicated high ESA dose or ESA resistance as the culprit for adverse cardiovascular events^18^,^19^,^20^. The United States Food and Drug Administration (FDA)^21^, the 2012 Kidney Disease: Improving Global Outcomes (KDIGO) guideline^22^, and the updated 2015 National Institute for Health and Care Excellence (NICE) guideline^23^ suggested to limit the upper Hb target to 11~12 g/dL and avoid normalization of Hb level. As a result, the use of ESAs, the mean ESA dose, and the mean Hb level in pre-dialytic CKD patients in America all decreased after 2007^24^,^25^. However, the effect of restricted ESA use on renal outcome in the contemporary era has not been well studied.

In Taiwan, the reimbursement of ESA is regulated by the National Health Insurance (NHI) program which has a coverage of up to 99% for the whole population. NHI program restricts the use of ESA in CKD patients with serum creatinine level of more than 6 mg/dL and hematocrit (Hct) level of less than 28%. Besides, the maximal monthly dose is limited to 20,000 U of epoetin beta or equivalent doses of other ESAs. According to the United States Renal Data System 2014 annual data report, Taiwan had the highest prevalence and took the second place in the incidence of ESRD^26^. In contrast, cardiovascular mortality in Taiwanese patients before dialysis was not as high as in the western countries^1^,^27^. We are intrigued by the possible impact of restricted ESA use regulated by NHI. Our group has previously shown that both multidisciplinary care program (MDCP)^28^ and pentoxifylline^29^ may reduce the risk for dialysis initiation in patients with advanced CKD. We aimed to study the association between ESA use and dialysis initiation in a time-dependent Cox regression model in a cohort of stage 5 CKD patients^30^.

## Results

### Baseline Characteristics of Patients and ESA Administration

**Figure 1:**
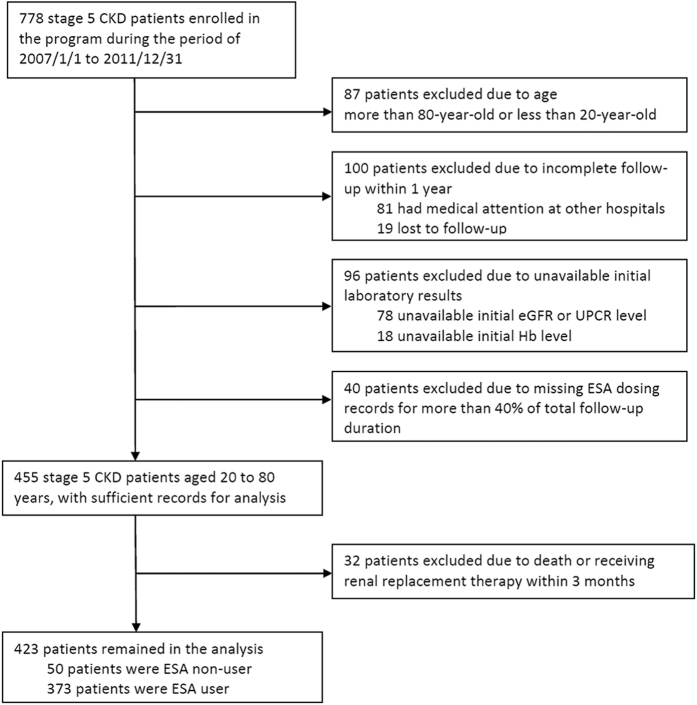
Flow diagram for the selection of patients in the analysis. Patients were excluded if the age, follow-up, laboratory record, or medication record criteria could not be fulfilled. Patients who reached death or received renal replacement therapy in less than 3 months were also excluded. Renal replacement therapy includes hemodialysis, peritoneal dialysis, and renal transplantation. ESA non-user was defined as not receiving any ESA during follow-up, while ESA user as receiving ESA in any given month. Abbreviations: CKD, chronic kidney disease; eGFR, estimated glomerular filtration rate; ESA, erythropoiesis-stimulating agent; Hb, hemoglobin; UPCR, urine protein-creatinine ratio.

We identified 423 stage 5 CKD patients fulfilling the inclusion/exclusion criteria in our cohort (Fig. 1). The median and interquartile range (IQR) of follow-up duration was 1.37 and 0.77–2.18 years, respectively. Totally 9,270 patient-months were analyzed. Patients who did not have any documented ESA administration during the entire study period were defined as ESA non-users, while those with ESA administration in any given period during the study were ESA users. The percentage of missing data in the monthly ESA dose was 4.1%. The baseline characteristics of patients stratified by ESA users and non-users at the time of enrollment into MDCP were listed in Table 1. Compared with ESA non-users, ESA users had lower levels of baseline estimated glomerular filtration rate (eGFR) and Hb, higher serum levels of phosphate and potassium, indicating more advanced CKD at the time of their entry into the study. Notably, the comorbidity of diabetes mellitus (DM) and hypertension were highly prevalent in this cohort. The levels of mean arterial pressure (MAP) and glycated hemoglobin (HbA1C) were well controlled in both groups. During the follow-up period, totally 290 patients (68.6%) initiated dialysis (187 hemodialysis and 103 peritoneal dialysis), 13 died (3.1%) and 8 received renal transplantation (1.9%). Among patients initiating dialysis, 73.2% were ESA user and 34% were ESA non-user. The crude rate of death among ESA users and ESA non-users were 2.4% and 8.0%, respectively (P = 0.06, Fisher’s exact test).

### The Levels and Annual Decline Rates of Hb and eGFR in ESA Users and Non-users

The levels and annual decline rates of Hb and eGFR in ESA users and non-users were summarized in Table 2. The median durations of follow-up were similar between ESA users and non-users. The standardized monthly ESA dose was 16,000 ± 3,900 U of epoetin beta during ESA treatment. Because the reimbursement of ESA use was regulated by NHI program, the average of standardized monthly ESA dose was 9,600 ± 5,500 U of epoetin beta during the study period including the months with and without ESA use. Compared to the baseline Hb level, there was no significant change of the Hb level overall in the last follow-up of ESA users. However, the annual change of Hb was −0.29 ± 2.19 g/dL/year in ESA users, which was less than that, −0.99 ± 2.46 g/dL/year, in ESA non-users (P = 0.038). Although the baseline and last eGFR levels were significantly lower in ESA users, the annual decline rates of eGFR, 3.45 ± 4.08 and 2.29 ± 5.12 mL/min/1.73 m^2^/year in ESA users and non-users respectively, were not different (P = 0.07). However, the median eGFR levels obtained before dialysis initiation were significantly lower in ESA users (ESA users 4.67 (3.63–5.67) ml/min/1.73 m^2^, ESA non-users 5.63 (4.63–7.62) ml/min/1.73 m^2^, P = 0.02).

### Association between ESA Use and Dialysis Initiation in a Time-dependent Cox Regression Model

Because the ESA dose, Hb and eGFR levels varied with time and the dosage of ESA was adjusted by Hb level in any given month according to the regulation of reimbursement agency, we constructed a multivariate time-dependent Cox regression model to investigate the association between ESA use and dialysis initiation (Table 3). Monthly ESA use, Hb and eGFR levels were incorporated as time-dependent variables. Variables significantly associated with dialysis initiation in the univariate analysis (P < 0.05) as well as variables deemed clinically relevant, which included age, sex, smoking status, use of Renin-Angiotensin-Aldosterone System (RAAS) blockade, monthly eGFR level, monthly Hb level, MAP, body mass index (BMI), primary glomerular disease (as an etiology for CKD), DM (as a comorbidity), ischemic heart disease (as a comorbidity), log urine protein-creatinine ratio (UPCR), and serum uric acid level, were forced into the multivariate Cox regression model (Supplementary Tables S1 and S2). Although ESA use was associated with increased hazard ratio (HR) of dialysis initiation in the univariate analysis, it was associated with decreased HR after multivariate adjustment (HR 0.63, 95% confidence interval (CI) 0.42–0.93, P = 0.021). The adjusted survival plot also supported the beneficial association (Supplementary Figure S1). When time-varying ESA use (use versus non-use) was replaced by time-varying monthly ESA dose (per 2,000 U/month) to analyze the association with dialysis initiation, the results were also similar (HR 1.08, 95% CI 1.06–1.11, per 2,000 U increment, P < 0.001, in univariate analysis, and HR 0.95, 95% CI 0.91–0.98, per 2,000 U increment, P = 0.004, after multivariate adjustment).

### Sensitivity Analysis and Subgroup Analysis

To test the robustness of the results and the heterogeneity among subgroups, we performed several pre-defined sensitivity analyses and subgroup analyses. Table 4 displayed the summary of sensitivity analyses. In short, changing the outcome definition from dialysis initiation to composite outcome (dialysis and death), changing the variable ESA use to ESA dose groups, adopting new ESA dose conversion formula^31^, changing eGFR estimation formula^32^,^33^, replacing MAP with systolic blood pressure (SBP) or diastolic blood pressure (DBP), changing BMI value to BMI groups^34^, and changing criteria for patient selection, all yielded similar HR and 95% CI.

Notably, when variable time-dependent eGFR was replaced with time-independent eGFR (baseline eGFR level), ESA use or ESA dose were associated with neutral or even harmful effects on dialysis initiation. However, replacing variable time-dependent ESA or Hb variable only with time-independent ESA (ESA user/ESA non-user or average ESA dose for each patient) or Hb (baseline Hb level) did not alter the beneficial results.

**Figure 2:**
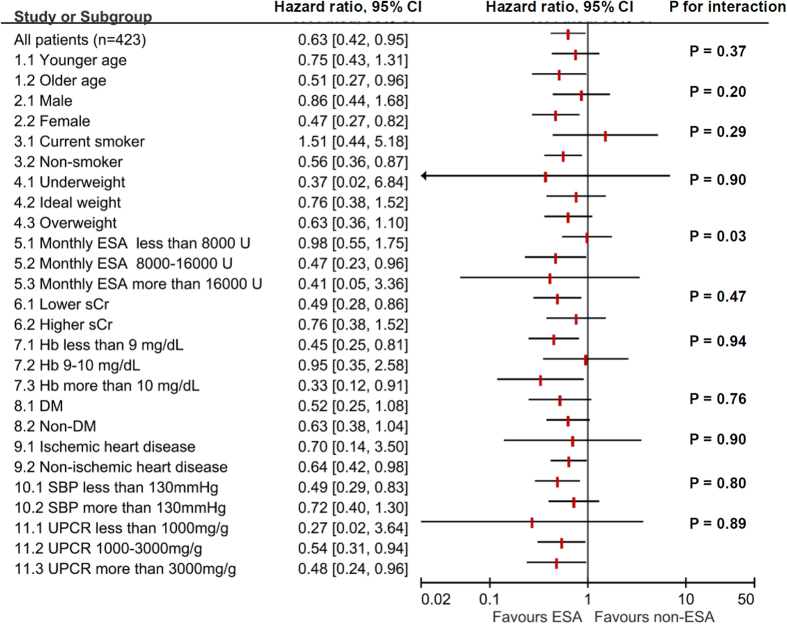
Subgroup analysis: ESA use and dialysis initiation. Definition for subgroups: 1. Age < 65-year-old denotes younger age. 3. Non-smoker includes ex-smoker. 4. The BMI cut-off points follow WHO suggestion in Asian population. 6. Serum creatinine level < 6 mg/dL denotes lower serum creatinine. 8. DM as a comorbidity. 9. Ischemic heart disease as a comorbidity. Abbreviation: BMI, body mass index; DM, diabetes mellitus; WHO, World Health Organization; SBP, systolic blood pressure; sCr, serum creatinine.

**Figure 3:**
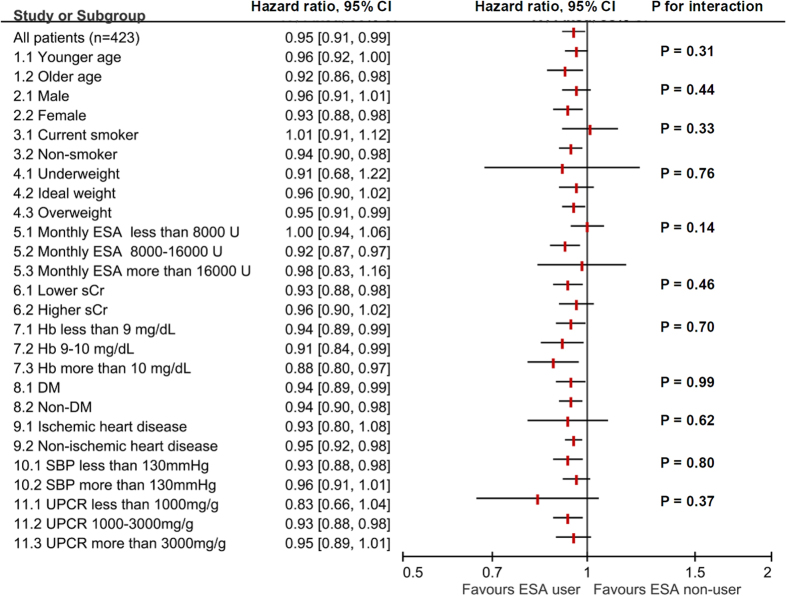
Subgroup analysis: ESA dose and dialysis initiation.

The effect of ESA use and ESA dose in subgroups were summarized in Figs 2 and 3. The favorable effect of ESA was consistent in different subgroups stratified by age, sex, BMI, serum creatinine levels, Hb levels, DM (as a comorbidity), ischemic heart disease (as a comorbidity), SBP levels, and UPCR levels. Interestingly, the test for interaction was significant in the subgroups of different standardized monthly ESA doses (5.1, 5.2, 5.3) in the ESA use model.

## Discussion

Two possible mechanisms can be envisaged to explain the association between ESA use and deferred dialysis initiation. First, ESA use confers renoprotective effect and retards deterioration of renal function. Second, ESA use improves general condition and tolerance to uremia, thereby deferring the need of dialysis initiation for uremic symptoms.

The renoprotective effects of EPO in both acute kidney injury (AKI) and CKD animal models have been widely studied^7^,^8^,^9^,^10^,^11^,^12^,^13^,^35^,^36^,^37^,^38^,^39^,^40^,^41^. In CKD models, the protective effects are linked with low-dose EPO rather than high-dose EPO^37^,^38^,^39^. Bahlmann FH and colleagues showed that the administration of low-dose darbepoetin, as low as not to affect the Hb level, improved survival and renal function in rats undergoing 5/6 nephrectomy^7^. Menne J and colleagues demonstrated that the protective effects of continuous erythropoietin receptor activator (CERA) in diabetic nephropathy were lost when the dose was increased to an extent of elevating Hct level^8^. In line with these findings, studies had shown that correction of anemia with high-dose recombinant human EPO in remnant kidney model resulted in hypertension and deterioration of renal function, possibly through activation of endothelin and renin-angiotensin system^37^,^38^,^39^. Importantly, the tissue protective effects of EPO and the erythropoiesis effects of EPO may involve different receptors and downstream signals. Recent studies have shown that βcR was essential for tissue protection and Klotho may enhance the effect^11^,^40^. A heterodimer receptor composed of βcR and EPO receptor (EPOR), rather than traditional EPOR homodimer, was proposed to mediate the tissue protective effects of EPO. It has also been shown that mice with βcR null mutation did not have significant abnormality in hematopoiesis^13^. To apply the basic findings in EPO related clinical trials, care should be exercised that correction of anemia does not ensure tissue protection, while failure to increase Hb level is not equal to loss of tissue protection. Investigators from the Correction of Hemoglobin and Outcome in Renal insufficiency (CHOIR) trial reported, despite adjustment of achieved Hb level, an average epoetin alfa dose of more than 10,095 U/week was associated with increased cardiovascular events. Besides, in patients who received ESA at a median weekly dose of 4,513 U, the relationship between dose and HR for adverse outcome appeared to be J shaped, and a weekly dose of 4,000 to 6,000 U was associated with the lowest risk^18^,^42^. Interestingly, ESA use was associated with renoprotection when ESA was administered in the lower dose range of 3,000 to 7,000 U per week in some small trials^14^,^15^,^43^.

Inspired by the aforementioned basic and clinical research, our study was designed to exam the possible beneficial effects of restricted ESA use in our non-dialysis cohort of stage 5 CKD patients with maximal standardized monthly dose of ESA equivalent to epoetin beta 20,000 U. In correspondence to the renal benefits of ESA shown in these clinical trials^14^,^15^,^43^, our data showed that dose of ESA equivalent to epoetin beta 16,000 U (~4,000 U/week) was safe and associated with deferral of dialysis initiation compared with no ESA use. Besides, every 2,000 U/month increment of the dose was significantly associated with deferred dialysis initiation, too. Although the dose of ESA used in this cohort did not increase Hb level, it attenuated the annual Hb decline significantly. It was hard to conclude that the deferral of dialysis initiation in ESA users was due to renoprotective effects of ESA because of the limitations inherent in the cohort study and the indiscriminate eGFR decline rates between ESA users and non-users. However, it is noteworthy that ESA users in our cohort presented lower baseline eGFR and Hb levels, but they experienced indifferent annual eGFR decline and deferred dialysis initiation when compared with ESA non-users. To delineate renoprotective effects of ESA, other study designs such as RCT comparing the effect of fixed ESA doses on CKD progression should be considered.

The second possible mechanism underlying the deferral of dialysis initiation by ESA use was the improvement of general condition and tolerance to uremia due to attenuated Hb decline. More severe anemia is generally associated with the lowest quality of life (QoL). Observational studies have shown that stage 5 CKD patients have the lowest levels of plasma EPO and Hb^44^,^45^, and will theoretically benefit most from ESA therapy. In our cohort of solely stage 5 CKD patients, the baseline Hb level was 9.3 ± 1.5 g/dL and 10.4 ± 1.8 g/dL in ESA users and non-users, indicating at least moderate severity of anemia. A significant decline of Hb level was observed in ESA non-users but not in ESA users. Evidence has shown that progressive anemia in dialysis or advanced CKD patients is associated with poor QoL and exercise tolerance, and ESA use can correct anemia and improve QoL as well^46^,^47^,^48^. Therefore we believe ESA use might improve general condition and tolerance to uremic symptoms in patients with advanced CKD and anemia, thereby deferring the need for dialysis initiation. In our cohort, the observed lower eGFR levels at dialysis initiation in ESA users than in ESA non-users may further support this hypothesis.

In this study, the crude overall mortality rate and dialysis rate were 3.1% (2.2 per 100 patient-years) and 68.6% (50 per 100 patient-years), respectively, which were comparable to our previous study^27^. Compared to the higher overall mortality rate in the western countries such as the United States (crude mortality rate 10.7 per 100 patient-years for stage 4 and 5 CKD patients)^26^, the lower mortality rate in our patients might have underlying genetic or environmental basis which needs further study. It is noteworthy that advanced CKD patients in Japan also presented with low mortality rate (crude mortality rate 2.0 per 100 patient-years for stage 4 and 5 CKD patients)^49^. In Japan, the maximal ESA dose is limited to 6,000 U epoetin-equivalent dose per week in pre-dialytic CKD patients^50^, which is comparable to the Taiwan NHI regulated maximal dose of 20,000 U per month. According to a recent report from Dialysis Outcomes and Practice Patterns Study (DOPPS), Japan had the lowest prescribed ESA dose (~5,509 U of epoetin-equivalent dose per week) compared with Europe (~8,744 U per week) and the United States (~15,784 U per week) from 2010 to 2013. The dose of ESA in dialysis patients in Taiwan may be even lower than in Japan, because Taiwan NHI restricts the ESA use in dialysis patients by the same regulation as in pre-dialytic CKD patients and the maximal dose is 20,000 U per month. Interestingly, according to DOPPS and dialysis registry among different countries, dialysis patients in Taiwan and Japan also had lower mortality rates compared with in western countries^26^,^51^,^52^,^53^,^54^. Although mortality rate in CKD patients is multifactorial and our study is not designed for analysis of the effect on mortality, our data demonstrated that ESA use lower than the dose equivalent to epoetin beta 20,000 U was safe and associated with deferral of dialysis initiation in stage 5 CKD patients.

In our cohort, the overall median of eGFR at dialysis initiation was 4.73 ml/min/1.73 m^2^ (IQR, 3.66–5.75) despite notably high prevalence of the comorbidities including DM and hypertension, which was similar to the data of 4.7 ml/min/1.73 m^2^ (IQR, 3.6–6.1) reported previously based on a national database in Taiwan^55^. Although the timing of dialysis initiation may be relative late, the survival of dialysis patients in Taiwan is comparable to Japan and better than many countries such as the United States^26^,^51^,^52^,^53^,^54^. Studies have also shown that early initiation does not provide survival benefit, and may even increase mortality risk in patients without significant comorbidity^56^,^57^,^58^. As a result, even in stage 5 CKD patients, effective interventions, such as restricted ESA use, to defer the need for dialysis initiation may still confer clinical and economic benefits, and should not be ignored.

Considering the time-varying nature of eGFR levels, Hb levels and ESA doses, our analyses using a time-dependent Cox regression model adjusted for time-dependent eGFR, Hb, and ESA variables. Importantly, replacing the time-varying eGFR variable to time-independent baseline eGFR variable would lead to a completely different effect of ESA use and dose on renal outcome. Since eGFR is an important predictor of renal outcome^59^,^60^ and it usually progressively declines with time, failure to incorporate its time-varying nature in the Cox regression may incur bias. Moreover, our data showed that ESA users had lower levels of baseline eGFR, suggesting more advanced CKD at the time of their entry and the potential of more rapid disease progression. Therefore, the model considering the eGFR changes was more appropriate than that ignoring the eGFR changes. Correspondingly, the time-dependent Cox regression model adjusted for time-varying Hb level and inflammatory parameters has been successfully used by Regidor DL and colleagues to study the beneficial effects of ESA on survival of hemodialysis patients^61^. Their data showed that patients receiving ESA of 1–5,999 U/week had less mortality hazard than patients not, while ESA dose of more than 6,000 U/week was associated with increased mortality hazard than the dose of 1–5,999 U/week.

The importance of competing risk of death had been demonstrated in prior studies including patients with all the 5 stages of CKD patients. The competing risk was especially high in CKD stage 1, 2, and 3^62^. However, in our cohort of solely stage 5 CKD patients, only 13 patients died in contrast to 290 patients who transited to stage 5D, in a median follow-up duration of less than 2 years. The competing risk is low and thus we did not apply competing risk analysis in our cohort. To further confirm the negligible impact of death, we excluded the 13 patients died during follow-up in the sensitivity analysis, and the result was reassuring similar.

In our subgroup analyses, statistical significance for heterogeneity was observed in subgroups of different standardized monthly ESA doses in the ESA use model, which may imply different effects on dialysis initiation with different monthly ESA doses. In the full model estimating the association between ESA dose and renal outcome, every 2,000 U increment of ESA dose was associated with better outcome. Furthermore, in our sensitivity analyses, when the variable of ESA use was changed to variables of different ESA dosing categories, the most significant and strongest beneficial association was observed in the category of highest monthly ESA dose (ESA > 16,000 U/month). In contrast to previous studies showing the harmful effects of high dose ESA, results from our study may imply that when limiting ESA dose to no more than 20,000 U/month, higher ESA dose may be associated with better renal outcome. However, it is unknown whether these observed beneficial associations still exist in ESA dose higher than 20,000 U/month. Future RCTs designed to test effects of different ESA doses are warranted to clarify these questions.

There were several limitations in our analyses. First, the nature of retrospective design precluded confirmation of causal relationship. The results of the analyses may be helpful for the hypothesis generation on ESA dose and renal outcome, but not to be viewed as a solid proof for the beneficial effects of ESA on renal outcome. Second, our data showed that eGFR obtained before dialysis initiation was significantly lower in ESA users. Thus the effect of ESA to defer dialysis initiation may be related to the improvement of general condition and uremia tolerability rather than true renoprotection. Besides, the calculation of annual eGFR decline rate in our analysis might be misleading, because the trajectory of renal function in most CKD patients had been shown to be non-linear^63^.

In conclusion, monthly ESA use lower than the equivalent dose of epoetin beta 20,000 U was safe and associated with deferral of dialysis initiation in stage 5 CKD patients despite lower baseline levels of eGFR and Hb in a time-dependent Cox regression model. However, retrospective design and lack of discernible effect on eGFR decline rate weakened the strength of the result. This study provides reference to the future RCTs designed to test the effects of different ESA doses on CKD progression and survival.

## Methods

### Patients

This was a single center retrospective cohort study and approved by the Research Ethics Committee at National Taiwan University Hospital (NTUH) (201405016RIND). The study was conducted on an encrypted database, and waiver of inform consent was approved by the committee. Patients enrolled in the MDCP between January 2007 and December 2011 were included in the analysis if they were between 20 to 80 years old and at CKD stage 5 defined by Kidney Disease Outcomes Quality Initiative (KDOQI) guideline^60^. The MDCP is initiated by the Ministry of Health and Welfare of Taiwan government in 2006, and the aim of the program is to reduce the high incidence and prevalence of ESRD in Taiwan^28^,^29^. Patients were excluded if they lost to follow-up or sought medical attention at other hospital within 1 year, had unavailable initial eGFR, Hb, or UPCR level, had unavailable ESA dosing record for a period of more than 40% of total follow-up duration, or received dialysis, renal transplantation or died within 3 months. The timing of dialysis initiation was decided by the primary care nephrologist. Uremic symptoms, refractory fluid overload, and refractory acid-base or electrolytes imbalance were the most common indications for initiation of dialysis.

The date of enrollment in the MDCP was defined as cohort entry date for each patient. Demographic characteristics, use of RAAS blockade, primary etiology of CKD, and comorbidities were recorded at the time of enrollment and were viewed as baseline information. The primary etiology of CKD was denoted by the primary care nephrologist. The definition for comorbidity was specified as the following: patients with a level of HbA1C >6.5%, fasting glucose >126 mg/dL, or using any oral anti-diabetic agents, insulin or insulin analogs were defined as having DM; patient with a level of SBP >140 mmHg or taking any anti-hypertensive agents were defined as having hypertension; the diagnoses of ischemic heart disease, stroke, malignancy, dyslipidemia, and gout were assessed by the primary care nephrologist. Ischemic heart disease included coronary artery disease and congestive heart failure.

### ESA Administration

The use of ESA in this study complied with the regulation of the NHI program in Taiwan. The NHI program reimbursed the ESA therapy when a patient had a serum creatinine level of more than 6.0 mg/dl and a Hct level of less than 28%. The maximal monthly doses of epoetin beta, darbepoetin alfa and methoxy polyethylene glycol-epoetin beta are 20,000 U, 100 μg and 100 μg, respectively. A maintenance target Hct level of 33–36% was suggested though often not achieved. The complete ESA dosing for each patient during follow-up period were extracted from electrical medical record (EMR) at NTUH. During the study period, three kinds of ESA were used, including epoetin beta, darbepoetin alfa, and methoxy polyethylene glycol-epoetin beta. The doses of methoxy polyethylene glycol-epoetin beta and darbepoetin alfa were converted to standardized equivalent doses of epoetin beta according to the WHO daily-defined dose (DDD) (methoxy polyethylene glycol-epoetin beta 4 μg or darbepoetin alfa 4.5 μg was equal to epoetin beta 1,000 U)^31^. Monthly ESA dose was calculated and recorded as a time-dependent variable for each patient. Patients who received ESA in any given month during study period were defined as ESA users. Patients who did not receive any ESA during the whole study period were defined as ESA non-users. In ESA users, the monthly administered ESA dose was estimated by averaging total ESA doses of each patient by the total months with ESA use.

### Laboratory Tests

All the laboratory tests were performed with standardized and automatic method at NTUH. Serum creatinine level was analyzed by modified Jaffe method with modification of alkaline picrate kinetics (Beckman Coulter AU analyzer, California, USA). The complete laboratory results for serum creatinine and Hb levels were extracted from EMR. The eGFR was estimated by Simplified Modification of Diet in Renal Disease (MDRD-S) equation^32^. Monthly eGFR and Hb levels were recorded as time-dependent variables for each patient. On the other hand, only baseline levels of UPCR, serum albumin, HbA1C, total cholesterol, calcium, phosphate, sodium, potassium, and uric acid were recorded. These laboratory parameters were expressed as time-independent variables. The last Hb and eGFR levels were the last available levels during follow-up. The eGFR level at dialysis initiation was the last available eGFR level before dialysis initiation. The annual eGFR decline rate for each patient was defined as the difference between the last eGFR and the initial eGFR levels divided by the follow-up duration of the patient. The annual Hb change rate was estimated similarly.

### Statistical Analysis

Multivariate time-dependent Cox regression models were constructed to assess the association between ESA administration and renal outcome. The outcome variable was initiation of dialysis, which included hemodialysis and peritoneal dialysis. Patient received renal transplantation or died before dialysis was censored. The exposure variable was ESA administration. We established two models according to ESA administration status. In the first model (ESA use model), the monthly ESA use status (yes vs. no) was a time-varying dichotomous variable. In the second model (ESA dose model), the monthly ESA dose was a time-varying continuous variable. If a patient had multiple medical records within a month, monthly average levels of ESA dose, eGFR, and Hb were used in analyses. The monthly eGFR and Hb levels were also time-varying variables. The covariates included age, sex, smoking status (current smoker or non-smoker), use of RAAS blockade, eGFR level, Hb level, MAP level, BMI, primary glomerular disease (as primary etiology for CKD), DM (as a comorbidity), ischemic heart disease (as a comorbidity), log transformed UPCR level, and uric acid level. We considered these covariates in the multivariate models either because of their clinical relevance to dialysis initiation or because of their statistically significant associations with dialysis initiation in univariate analyses.

An important assumption in the Cox regression model is that the effect of any predictor variable is constant over time^30^. This examination was performed with PROC PHREG in SAS 9.2 (Cary, North Carolina, USA) by creating time-varying covariates and using the “proportionality test” statement^64^. We checked the proportional hazard assumption for the two multivariate Cox regression models in Table 3. The P values of the proportionality tests were 0.9199 and 0.9493 for the ESA use full adjustment model and the ESA dose full adjustment model, respectively. These non-significant proportionality test results suggested the proportional hazard assumption held for our models.

Sensitivity analyses were performed to test the robustness of the models. First, we changed the definition of outcome variable in the original model to test the consistency of the results. Next, we replaced one of the exposure variable or covariates with a new relevant variable one at a time. We also modified the criteria for patient selection. Finally, we replaced time-dependent variable(s) in the original time-dependent model with time-independent baseline variable(s), and found that the effect of ESA on renal outcome might change if the model did not incorporate the time-varying nature of eGFR.

Subgroup analyses were also performed to test the heterogeneity between subgroups. Subgroups of different age, sex, smoking status, BMI status, monthly ESA dose, serum creatinine level, Hb level, diabetes comorbidity, ischemic heart disease comorbidity, SBP level, and UPCR level were identified. In the subgroups of different ESA doses (subgroup 5.1, 5.2, 5.3), patients were included in the specific subgroup according to the standardized monthly ESA dose. In the subgroups of different Hb levels (7.1, 7.2, 7.3), data were stratified according to time-varying monthly Hb levels. In other subgroups, patients were stratified according to baseline characteristics. HRs within each subgroup were estimated with the defined multivariate time-dependent Cox regression model adjusted for age, sex, smoking status, use of RAAS blockade, eGFR level, Hb level, MAP level, BMI, primary glomerular disease (as primary etiology for CKD), DM (as a comorbidity), ischemic heart disease (as a comorbidity), log transformed UPCR level, and uric acid level. In tests for interaction, we added an interaction product variable “subgroup*ESA” in the multivariate model to determine the P value for interaction.

Continuous variables were presented as mean ± standard deviation or median (IQR), and the difference was compared with the Student’s *t*-test or the nonparametric Mann-Whitney *U* test, whichever appropriate. Categorical variables were presented as percentages and analyzed with the chi-square test or Fisher’s exact test, whichever appropriate. Statistical significance was claimed if P < 0.05. All these statistical analyses were performed with the PHREG procedure in SAS 9.2 (Cary, North Carolina, USA).

## Additional Information

**How to cite this article:** Pan, S.-Y. *et al*. Restricted Use of Erythropoiesis-Stimulating Agent is Safe and Associated with Deferred Dialysis Initiation in Stage 5 Chronic Kidney Disease. *Sci. Rep.* **7**, 44013; doi: 10.1038/srep44013 (2017).

**Publisher's note:** Springer Nature remains neutral with regard to jurisdictional claims in published maps and institutional affiliations.

## Supplementary Material

Supplementary Information

## Figures and Tables

**Table 1:** Baseline characteristics of stage 5 CKD patients stratified by ESA user and non-user.

Characteristic	ESA user	ESA non-user	P value
	N = 373	N = 50	
Age (year)	60 ± 12	62 ± 13	0.27
Male sex (%)	43%	42%	0.88
MAP (mmHg)	96 ± 11	98 ± 12	0.14
Smoker (%)	17%	26%	0.10
BMI (kg/m^2^)	24 ± 4.7	25 ± 3.6	0.06
Use of RAAS blockade	26%	40%	0.04
Primary etiology for CKD			
Primary glomerular disease (%)	39%	36%	0.67
Diabetes mellitus (%)	30%	30%	0.99
Hypertension (%)	8%	2%	0.15
Obstructive nephropathy (%)	2.4%	6%	0.16
Polycystic kidney disease (%)	3.5%	4%	0.69
Unknown (%)	11%	14%	0.53
Comorbidity			
Diabetes mellitus (%)	38%	50%	0.11
Hypertension (%)	66%	64%	0.81
Ischemic heart disease^a^ (%)	11%	14%	0.53
Stroke (%)	2.7%	6%	0.19
Malignancy (%)	4%	8%	0.26
Dyslipidemia (%)	15%	20%	0.31
Gout (%)	8%	14%	0.19
Laboratory parameters			
eGFR (mL/min/1.73 m^2^)	9.4 ± 2.8	12.0 ± 2.6	<0.01
Hb (g/dL)	9.3 ± 1.5	10.4 ± 1.8	<0.01
Albumin (g/dL)	4.2 ± 0.4	4.4 ± 0.5	0.06
Log UPCR (mg/mg)	3.3 ± 0.4	3.3 ± 0.4	0.24
HbA1C (%)	6.7 ± 1.3	6.4 ± 0.8	0.22
Total cholesterol (mg/dL)	181 (152–216)	188 (161–207)	0.87
Calcium (mmol/L)	2.2 ± 0.2	2.2 ± 0.3	0.69
Phosphate (mg/dL)	4.9 ± 1.1	4.4 ± 0.9	<0.01
Sodium (mmol/L)	138 ± 4	138 ± 4.0	0.34
Potassium (mmol/L)	4.8 ± 0.7	4.5 ± 0.7	0.01
Uric acid (mg/dL)	8.5 ± 1.9	8.7 ± 2.5	0.53

Continuous variables were presented as mean ± s.d. or as median (interquartile range). Difference of continuous variables between ESA user and non-user were compared with the Student’s *t*-test or the nonparametric Mann-Whitney *U* test. Categorical variables were presented as percentages and analyzed with the chi-square test or Fisher’s exact test. ^a^Ischemic heart disease includes coronary artery disease and congestive heart failure.

Abbreviation: BMI, body mass index; CKD, chronic kidney disease; eGFR, estimated glomerular filtration rate; ESA, erythropoiesis-stimulating agent; Hb, hemoglobin; HbA1C, glycated hemoglobin; MAP, mean arterial pressure; RAAS, Renin-Angiotensin-Aldosterone System; UPCR, urine protein-creatinine ratio.

**Table 2:** The levels and annual decline rates of Hb and eGFR in ESA user and non-user.

	ESA user	ESA non-user	P value
	N = 373	N = 50	
Standardized monthly ESA dose (U/month/patient)	16,000 ± 3,900	0	
Duration of follow-up (year)	1.36 (0.72–2.17)	1.44 (1.15–2.37)	0.22
Hb			
Baseline Hb level (g/dL)	9.3 ± 1.5	10.4 ± 1.8	<0.01
Last Hb level (g/dL)	9.1 ± 1.4	9.7 ± 1.8	0.02
Annual decline rate of Hb level (g/dL/year)	0.29 ± 2.19	0.99 ± 2.46	0.04
eGFR			
Baseline eGFR level (mL/min/1.73 m^2^)	9.4 ± 2.8	12.0 ± 2.6	<0.01
Last eGFR level (mL/min/1.73 m^2^)	5.5 ± 2.9	10.4 ± 5.7	<0.01
Annual decline rate of eGFR (mL/min/1.73 m^2^/year)	3.45 ± 4.08	2.29 ± 5.12	0.07

The standardized monthly ESA dose was the total exposed ESA dose divided by the duration of each patient with ESA use. The baseline Hb and eGFR levels were obtained at the time of enrollment as in Table 1. The last Hb and eGFR levels were the last available levels during follow-up. The annual decline rate of Hb level was the difference of baseline Hb level and last Hb level divided by follow-up duration of each patient. The annual decline rate of eGFR was the difference of baseline eGFR level and last eGFR level divided by follow-up duration of each patient.

**Table 3:** Association between ESA use and dialysis initiation in a time-dependent Cox regression model.

	Crude HR and 95% CI	P value^a^	Adjusted HR^b^ and 95% CI	P value^c^
ESA use	2.70 (1.96–3.73)	<0.001	0.63 (0.42–0.93)	0.021
ESA monthly dose (per 2,000 U/month)	1.08 (1.06–1.11)	<0.001	0.95 (0.91–0.98)	0.004

^a^P value for univariate analysis. ^b^Variables adjusted in the model: age, sex, smoking status, use of RAAS blockade, monthly eGFR level, monthly Hb level, MAP, BMI, primary glomerular disease (as an etiology for CKD), diabetes mellitus (as a comorbidity), ischemic heart disease (as a comorbidity), log UPCR level, and uric acid level. ^c^P value for multivariate analysis.

Abbreviation: CI, confidence interval; HR, hazard ratio.

**Table 4:** Sensitivity analysis.

	Variable(s) to be replaced	New variable	HR and 95% CI for ESA use	P value^a^	HR and 95% CI for ESA dose (per 2000 U/month)	P value^b^
Original model (n = 423)	Nil	Nil	0.63 (0.42–0.93)	0.021	0.95 (0.91–0.98)	0.004
Outcome variable	Initiation of dialysis^c^	Composite outcome^d^	0.62 (0.42–0.92)	0.017	0.95 (0.91–0.98)	0.002
Exposure variable	ESA use	ESA dose group^e^				
		<8000 U/month	0.78 (0.35–1.71)	0.530	NA	NA
		8000–1600 U/month	0.82 (0.48–1.40)	0.460	NA	NA
		>16000 U/month	0.56 (0.37–0.85)	0.006	NA	NA
	ESA dose conversion according to WHO DDD^f^	ESA dose conversion according to Taiwan NHI regulation^g^	NA	NA	0.94 (0.90–0.98)	0.002
Covariate	eGFR estimation with MDRD-S formula	eGFR estimation with CKD-EPI formula	0.62 (0.42–0.92)	0.017	0.95 (0.91–0.98)	0.003
	MAP	SBP	0.62 (0.42–0.91)	0.016	0.95 (0.91–0.98)	0.003
	MAP	DBP	0.61 (0.41–0.91)	0.015	0.95 (0.91–0.98)	0.003
	BMI	BMI groups^h^	0.62 (0.42–0.92)	0.016	0.95 (0.91–0.98)	0.003
Patient selection	Exclude patients received RRT or death within 3 months (n = 423)	Include patients received RRT or death within 3 months (n = 455)	0.67 (0.46–0.98)	0.036	0.95 (0.92–0.98)	0.003
	Include patients died after 3 months during follow-up (n = 423)	Exclude patients died after 3 months during follow-up (n = 410)	0.62 (0.42–0.91)	0.015	0.95 (0.91–0.98)	0.002
Time-dependent model	Time-dependent ESA, eGFR, and Hb	Time-independent ESA, eGFR, and Hb^i^	1.85 (1.02–3.38)	0.045	1.05 (1.00–1.11)	0.046
	Time-dependent eGFR	Time-independent eGFR^i^	1.38 (0.94–2.04)	0.101	1.01 (0.98–1.04)	0.600
	Time-dependent ESA	Time-independent ESA^i^	0.47 (0.26–0.86)	0.014	0.89 (0.85–0.93)	<0.001
	Time-dependent Hb	Time-independent Hb^i^	0.64 (0.43–0.95)	0.026	0.96 (0.92–0.99)	0.012
	Time-dependent eGFR and ESA	Time-independent eGFR and ESA^i^	1.18 (0.66–2.13)	0.570	0.98 (0.93–1.03)	0.350
	Time-dependent eGFR and Hb	Time-independent eGFR and Hb^i^	2.25 (1.51–3.35)	<0.001	1.06 (1.03–1.09)	<0.001
	Time-dependent ESA and Hb	Time-independent ESA and Hb^i^	0.50 (0.27–0.92)	0.025	0.89 (0.85–0.94)	<0.001

^a^P value for the hazard ratio of ESA use in the fully adjusted model. ^b^P value for the hazard ratio of ESA dose in the fully adjusted model. ^c^Event was defined as initiation of dialysis. Death or renal transplantation was censored. ^d^Event was defined as initiation of dialysis or death. Renal transplantation was censored. ^e^Monthly ESA dose of zero was set as reference group. The HRs of 3 different monthly ESA dose groups over reference group were estimated. ^f^Dose equivalent: 1000 U epoetin beta = 4.5 μg darbepoetin alfa = 4.0 μg methoxy polyethylene glycol-epoetin beta. ^g^Dose equivalent: 1000 U epoetin beta = 5.0 μg darbepoetin alfa = 5.0 μg methoxy polyethylene glycol-epoetin beta. ^h^BMI between 18.5–23 was set as reference group. The dummy variables of BMI < 18.5 and BMI > 23 over reference were incorporated in the model. ^i^The ESA user/ESA non-user variable was used as time-independent ESA use variable, and the standardized monthly dose of ESA of each patient was used as time-independent ESA dose variable. The baseline eGFR level was used as time-independent eGFR variable. The baseline Hb level was used as time-independent Hb variable.

Abbreviation: CKD-EPI, chronic kidney disease epidemiology collaboration; DBP, diastolic blood pressure; DDD, daily-defined dose; MAP, mean arterial pressure; MDRD-S, simplified modification of diet in renal disease; NHI, national health insurance; SBP, systolic blood pressure; WHO, world health organization.
