# Supplementary material for: Restricted Use of Erythropoiesis-Stimulating Agent is Safe and Associated with Deferred Dialysis Initiation in Stage 5 Chronic Kidney Disease
Source: Sci Rep. 2017 Mar 8;7:44013. doi: 10.1038/srep44013 (PMC5341043; doi:10.1038/srep44013)
Supplement: Supplementary Information [file srep44013-s1.pdf]

Pan et al. Supplementary information

Restricted Use of Erythropoiesis-Stimulating Agent is Safe and Associated with Deferred Dialysis Initiation in Stage 5 Chronic Kidney Disease

Szu-Yu Pan, Wen-Chih Chiang, Ping-Min Chen, Heng-Hsiu Liu, Yu-Hsiang Chou, Tai-Shuan Lai, Chun-Fu Lai, Yen-Ling Chiu, Wan-Yu Lin, Yung-Ming Chen, Tzong-Shinn Chu, Shuei-Liong Lin

Supplementary Table S1 Multivariate Cox regression model of ESA use and dialysis initiation

Variable	Crude HR and 95% CI	P value[a]	Adjusted HR[b] and 95% CI	P value[c]
ESA use	2.70 (1.96-3.73)	<0.001	0.63 (0.42-0.93)	0.021
Age (year)	0.99 (0.98-1.00)	0.058	1.01 (0.99-1.02)	0.385
Male sex	1.41 (1.12-1.78)	0.004	2.28 (1.60-3.23)	<0.001
Smoker	1.15 (0.86-1.55)	0.345	0.76 (0.48-1.19)	0.224
Use of RAAS blockade	0.77 (0.59-1.01)	0.057	1.15 (0.79-1.68)	0.469
eGFR (mL/min/1.73m^2)	0.63 (0.59-0.68)	<0.001	0.58 (0.53-0.65)	<0.001
Hb (g/dL)	0.73 (0.68-0.79)	<0.001	0.87 (0.78-0.97)	0.012
MAP (mmHg)	1.02 (1.01-1.03)	0.004	1.00 (0.98-1.01)	0.838
BMI (kg/m^2)	1.00 (0.97-1.02)	0.851	0.98 (0.94-1.01)	0.160
Primary glomerular disease	0.77 (0.61-0.98)	0.033	0.74 (0.49-1.12)	0.163
Diabetes mellitus (as a comorbidity)	1.31 (1.03-1.66)	0.026	0.94 (0.58-1.53)	0.815
Ischemic heart disease (as a comorbidity)	1.47 (1.04-2.08)	0.031	1.43 (0.86-2.39)	0.169
Log UPCR (mg/mg)	5.15 (3.55-7.46)	<0.001	3.26 (1.71-6.21)	<0.001
Uric acid (mg/dL)	1.14 (1.07-1.20)	<0.001	1.18 (1.07-1.29)	<0.001

[a]P value for univariate analysis. [b]Variables adjusted in the model: age, sex, smoking status, use of RAAS blockade, monthly eGFR level, monthly Hb level, MAP, BMI, primary glomerular disease (as an etiology for CKD), diabetes mellitus (as a comorbidity), ischemic heart disease (as a comorbidity), log UPCR level, and uric acid level. [c]P value for multivariate analysis.

Abbreviation: BMI, body mass index; CI, confidence interval; eGFR, estimated glomerular filtration rate; ESA, erythropoiesis-stimulating agent; Hb, hemoglobin; HR, hazard ratio; MAP, mean arterial pressure; RAAS, Renin-Angiotensin-Aldosterone System; UPCR, urine protein-creatinine ratio

Supplementary Table S2 Multivariate Cox regression model of ESA dose and dialysis initiation

Variable	Crude HR and 95% CI	P value[a]	Adjusted HR[b] and 95% CI	P value[c]
Monthly ESA dose (every 2000U increment)	1.08 (1.06-1.11)	<0.001	0.95 (0.91-0.98)	0.004
Age (year)	0.99 (0.98-1.00)	0.058	1.01 (0.99-1.02)	0.375
Male sex	1.41 (1.12-1.78)	0.004	2.34 (1.65-3.34)	<0.001
Smoker	1.15 (0.86-1.55)	0.345	0.75 (0.48-1.18)	0.216
Use of RAAS blockade	0.77 (0.59-1.01)	0.057	1.13 (0.77-1.65)	0.544
eGFR (mL/min/1.73m^2)	0.63 (0.59-0.68)	<0.001	0.58 (0.53-0.64)	<0.001
Hb (g/dL)	0.73 (0.68-0.79)	<0.001	0.86 (0.77-0.95)	0.004
MAP (mmHg)	1.02 (1.01-1.03)	0.004	1.00 (0.98-1.01)	0.810
BMI (kg/m^2)	1.00 (0.97-1.02)	0.851	0.98 (0.94-1.01)	0.146
Primary glomerular disease	0.77 (0.61-0.98)	0.033	0.75 (0.50-1.13)	0.166
Diabetes mellitus (as a comorbidity)	1.31 (1.03-1.66)	0.026	0.96 (0.59-1.55)	0.866
Ischemic heart disease (as a comorbidity)	1.47 (1.04-2.08)	0.031	1.44 (0.87-2.40)	0.160
Log UPCR (mg/mg)	5.15 (3.55-7.46)	<0.001	3.41 (1.78-6.54)	<0.001
Uric acid (mg/dL)	1.14 (1.07-1.20)	<0.001	1.18 (1.08-1.29)	<0.001

[a]P value for univariate analysis. [b]Variables adjusted in the model: age, sex, smoking status, use of RAAS blockade, monthly eGFR level, monthly Hb level, MAP, BMI, primary glomerular disease (as an etiology for CKD), diabetes mellitus (as a comorbidity), ischemic heart disease (as a comorbidity), log UPCR level, and uric acid level. [c]P value for multivariate analysis.

Supplementary Figure S1 Adjusted survival plot for ESA use and dialysis initiation

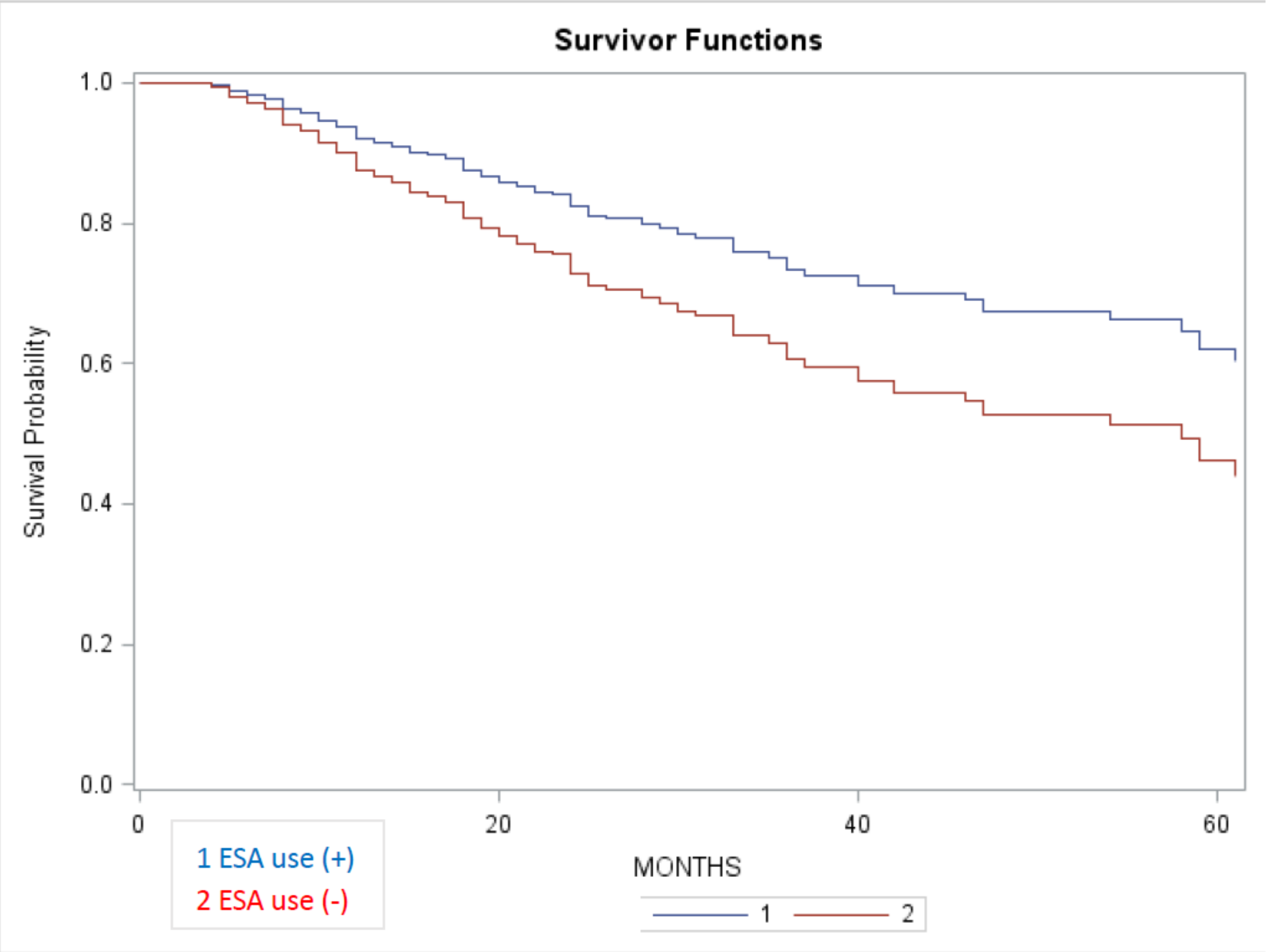

Variables adjusted in the model: age, sex, smoking status, monthly eGFR level, monthly Hb level, MAP, BMI, primary glomerular disease (as an etiology for CKD), diabetes mellitus (as a comorbidity), ischemic heart disease (as a comorbidity), log UPCR level, and uric acid level.
